# Solutions in microbiome engineering: prioritizing barriers to organism establishment

**DOI:** 10.1038/s41396-021-01088-5

**Published:** 2021-08-21

**Authors:** Michaeline B. N. Albright, Stilianos Louca, Daniel E. Winkler, Kelli L. Feeser, Sarah-Jane Haig, Katrine L. Whiteson, Joanne B. Emerson, John Dunbar

**Affiliations:** 1 Bioscience Division, Los Alamos National Laboratory, Los Alamos, NM USA; 2 Department of Biology, University of Oregon, Eugene, OR USA; 3 United States Geological Survey, Southwest Biological Science Center, Moab, UT USA; 4 Department of Civil and Environmental Engineering, University of Pittsburgh, Pittsburgh, PA USA; 5 Department of Molecular Biology and Biochemistry, University of California, Irvine, CA USA; 6 Department of Plant Pathology, University of California, Davis, CA USA

**Keywords:** Community ecology, Microbial ecology

## Abstract

Microbiome engineering is increasingly being employed as a solution to challenges in health, agriculture, and climate. Often manipulation involves inoculation of new microbes designed to improve function into a preexisting microbial community. Despite, increased efforts in microbiome engineering inoculants frequently fail to establish and/or confer long-lasting modifications on ecosystem function. We posit that one underlying cause of these shortfalls is the failure to consider barriers to organism establishment. This is a key challenge and focus of macroecology research, specifically invasion biology and restoration ecology. We adopt a framework from invasion biology that summarizes establishment barriers in three categories: (1) propagule pressure, (2) environmental filtering, and (3) biotic interactions factors. We suggest that biotic interactions is the most neglected factor in microbiome engineering research, and we recommend a number of actions to accelerate engineering solutions.

Microbiome engineering is a rapidly evolving frontier for solutions to improve human health, agricultural productivity, and climate management. Microbiome engineering seeks to improve the function of an ecosystem by manipulating the composition of microbes. Two major challenges for successful microbiome engineering are (1) the design of a microbiome with improved function and (2) the establishment of an improved microbiome in a recipient system of interest. While multiple articles and reviews have addressed functional design [1–3], microbiome establishment has received less attention. Here, we propose a strategy to improve microbiome engineering by focusing on microbial establishment and leveraging insights from macrobial ecology.

Two general engineering strategies are to manipulate indigenous microbes [4] or to introduce new members [5]. The latter involves the design and delivery of inoculants (a.k.a., probiotics in medical and agricultural arenas) and is a rapidly growing biotechnology sector. In their most general form, both strategies have been practiced crudely for thousands of years in human health [6] and agriculture [7]. However, despite current technical advances, inoculants frequently still fail to establish or confer long-lasting (months to years) modifications to ecosystem function [8]. We argue that this repeated failure is in part driven by lack of emphasis on establishment of inoculants.

The problem of organism establishment in recipient ecosystems is not unique to microbiome engineering; it has roots in macrobiology, particularly invasion biology and restoration ecology. We propose that adopting a cross-disciplinary conceptual framework to identify barriers to organism establishment, and then prioritizing these barriers through targeted research will accelerate successful microbiome engineering. In addition, recognizing differences in terminology and experimental design within and across disciplines will facilitate research integration across diverse ecosystems and scales. The components of a more holistic strategy are discussed below.

## Conceptual framework of barriers to organism establishment

Pinpointing and overcoming barriers to inocula establishment are important research priorities for successful microbiome engineering. Barriers to establishment have been studied extensively in both invasion biology and restoration ecology. Invasion biology aims to understand the mechanisms that promote or deter invasive species establishment [9]. Restoration ecology aims to restore beneficial plants, animals, insects, and/or microbial communities in ecosystems where they have previously been displaced or depleted [10]. Over time, these fields have produced numerous theoretical frameworks to explain successful species establishment [9, 11]. The overlapping foundational theory in these fields presents an opportunity for cross-disciplinary synergy with microbiome engineering. Although the goals of microbiome engineering and restoration/invasion ecology are often different (e.g., establishment of a new microorganism, consortium, and/or set of functional properties in microbiome engineering vs. prevention of establishment and/or removal of invading species and preservation of native biota in restoration/invasion ecology), the value in a cross-disciplinary approach to microbiome engineering that considers restoration/invasion ecology is in identifying emergent properties of the ecosystem that can facilitate or inhibit establishment. Furthermore, microbial ecology can advance foundational theory by overcoming common constraints on the scope and pace of macrobial ecology research, such as the relatively slow growth rates of macroscopic organisms and the difficulty of performing multi-species manipulations. Although microbial studies of factors influencing establishment are still relatively rare, foundational studies are emerging [12].

A useful theoretical framework for species establishment in microbiome engineering would identify the abiotic and biotic levers that engineers could use for successful community manipulation. Frameworks that vary in granularity have been developed within invasion biology. At one end of the spectrum, one synthesis outlined 33 mechanistic hypotheses for establishment success [13]. At the other end of the spectrum, a widely accepted framework posits that invasion success is mainly determined by three factors: (1) propagule pressure, (2) environmental filtering, and (3) biotic interactions [14]. Propagule pressure describes dispersal potential, which determines the spread of organisms to novel areas, either through natural- or human-mediated movement. Environmental filtering describes the compatibility of an organism with a new environment (e.g., suitable temperature or moisture range). Biotic interactions encompass a range of interchanges that can occur between introduced organisms and residents. Using this three-factor framework from invasion biology, in the next section we illustrate some complexity underlying these factors, illustrating the need for strategic prioritization as a first step to guide microbiome engineering (Table 1, Table S1).

**Table 1:** Overview of factors impeding organism establishment, potential engineering solutions. A comprehensive source of examples of studies which illustrate barriers to establishment and solutions is provided in Table S1.

Ecological principle	Factor impeding establishment	Potential engineering goal
Propagule pressure		
• Dose	**a**. Stochastic extinction **b**. Density-dependent competitiveness	**a**. Add a higher dose of the inoculant **b**. Add a lower dose of the inoculant
• Frequency	**a**. Succession makes niche ephemeral **b**. Biotic disturbance (i.e., inoculation) is needed to open a niche	**a**. Add inoculant more frequently **b**. Add inoculant at certain timepoint(s)
• Delivery mode	**a**. Inoculants do not reach or do not stay in intended location	**a**. Alter delivery mode **b**. Increase dose
Environmental filtering		
• Disturbance	High-turnover of organisms (low residence time)	**a**. Persistent delivery of inoculant is necessary **b**. Create a protected physical space **c**. Inoculant with characteristics resistant to disturbance
• Niche Breadth	Inoculated organism requires a specific resource that is absent	**a**. Engineer inoculant that has a larger niche breadth **b**. Pre-adapt inoculant to available environment **c**. Add resource specific to inoculant (‘pre-biotic’)
Biotic interactions		
• Antagonism via Competition	Direct competition exists between resident organisms and inoculants	**a**. Remove/disturb resident microbes **b**. Increase “competitive” trait of inoculation (e.g., antibiotic production, biofilm formation) **c**. Both a & b **d**. Add resources to support the inoculant during establishment period or beyond **e**. Pre-adapt inoculant to available environment **f**. Create a protected space
• Antagonism via Antibiotics	Antibiotic-producing residents debilitate the inoculant	**a**. Make inoculant resistant **b**. Disrupt resident(s) (reduce antibiotic(s) production) **c**. Create a protected space
• Antagonism via Predation	Predation by resident microbes	**a**. Make the inoculant resistant **b**. Remove predators prior to inoculation **c**. Create a protected space
• Facilitation	Inoculant requires ‘services’ provided by another organism which is not present	**a**. Add in an additional organism serving as a ‘keystone’ species to modify interactions of the target inoculant and other organisms and/or modify the environment

## Prioritizing barriers to establishment

### Potential barrier: propagule pressure

#### Dose and frequency

Propagule pressure (PP) is a measure of dispersal used to describe the magnitude (dose) and pattern of the arrival (frequency) of invasive individuals. PP is one of the most commonly tested factors in macroorganism invasion biology and is often linked to invasion success [15]. It also plays a role in restoration ecology [11]. For example, increased seeding rates can aid restoration of native plant communities (e.g., [16]), but is not always effective [17]. Increased PP of a single or a few microbial invaders may also increase establishment success [18]. However, independent modeling and experimental work suggest that PP in multi-species microbial invasions has restricted or minimal impacts on establishment and community functioning [19, 20].

There are a number of mechanisms by which PP in theory could influence establishment. Increasing PP can overcome the effects of ecological drift—random births and deaths that change the relative abundances of species in a community over time—also known as demographic stochasticity, which generally increases for smaller populations such as newly introduced species [21, 22]. Increasing PP can also mitigate the impacts of environmental stochasticity, unpredictable spatiotemporal fluctuations in environmental conditions [21]. A higher dose may increase the likelihood that sufficient inoculum reaches the desired establishment location [23] or may impact density-dependent competitiveness by affecting quorum sensing behaviors either positively or negatively [24]. The temporal frequency of dose events is an alternative route to enhance dosage while also addressing uncertain timing of niche access.

#### Delivery mode

Delivery mode may influence establishment by affecting dispersal range or dose viability. If the delivery mode is insufficient, inoculants may not reach the intended location of establishment or may be debilitated when they arrive. Parallels in plant invasion biology and restoration ecology include natural seed coats that enhance plant dispersal range by animal vectors [25] or artificial seed coatings that prevent desiccation or delay germination [26]. Delivery modes in microbiome engineering include direct addition of inoculants as free cells in liquids, lyophilized cells on solids (e.g., on animal feed or on seed surfaces), or protected cells (e.g., within seeds or gel beads) [27]. A delivery mode may be chosen to enhance the probability of microbial inoculant establishment based on environmental conditions and/or the traits of an inoculant [8].

### Potential barrier: environmental filtering

Environmental filtering (EF) refers to the selection of organisms that are compatible with the existing environment or are able to rapidly modify local conditions to fit requirements. Incompatible immigrants become extinct. EF can limit invasive plant establishment (e.g., [28]) and often explains restoration failures (e.g., [11]). Invasion success is expected to increase if invaded environments match those of a species optimum range [29]. In restoration, environmental filters are commonly manipulated to facilitate establishment of target restoration species [30] and/or prevent invader establishment [31]. An example of this phenomenon among microbes is the role of host specificity in microbial invasion success [32]. In both macro and microbiology, the most broadly successful invaders tend to have larger habitat ranges than non-successful invaders [33], which typically corresponds to tolerance of diverse conditions. EF conventionally includes abiotic (physical, chemical) and biotic factors (e.g., interactions with resident species). However, for engineering purposes it is useful to separate biological interactions (BI) from other modes of EF when considering barriers to establishment and potential solutions, because BI are generally harder to control for than abiotic factors. When EF is a barrier to microbiome engineering, either the expected longevity of engineering or the environmental conditions must be adjusted. Below, we summarize two aspects of EF to consider for microbiome engineering: niche availability and disturbance.

#### Niche availability

A lack of niche space for an inoculant is an obvious barrier to establishment. Understanding the temporal and spatial distributions of niche availability may be key to tailoring inoculant establishment [34]. Identification of a target niche space for inoculants through in situ strain characterization and subsequent isolation has led to success in a number of bioaugmentation efforts [35] and more recently, high-throughput phenotyping assays have been used [36]. If the target niche for an inoculant exists but is already filled, displacing the resident competitors may be required (e.g., [37]; see Biotic Interactions).

Alternatively, a niche can be created. Niche availability can be manipulated in some cases by altering the abundance of a single or multiple resource(s), as is done with prebiotics [4]. In host-associated communities, the host can provide a substrate to recruit health-promoting taxa, as illustrated by oligosaccharides in human breast milk that foster the growth of *Bifidobacterium infantis* strains in infant guts [38]. Similarly, there is increasing evidence that plant roots secrete metabolites to shape rhizosphere microbial composition [39]. Prebiotics can also be used to create novel niches for exotic inoculants, resulting in a combined pre- and probiotic known as a synbiotic. For example, porphyran—a marine polysaccharide—was used to establish an exogenous *Bacteriodetes* strain in mice guts [40] and xenobiotic compounds have been applied to support microbes of interest in industrial fermentations [41]. Continual delivery of an exotic resource can enable long-term stability of a “specialist” organism [42]. Conversely, short-term delivery of an exogenous resource may aid ‘generalist’ microbial inoculants that have a wider niche breadth but need a resource supplement during the transitional establishment period [43]. An alternative to manipulating niche availability is to exploit microbial inoculants that modify the environment, constructing their own niche [44] or a niche for other inoculants ([45], see Biotic Interactions).

#### Disturbance

Disturbance, here defined as perturbation of physical (e.g., structure, flow rates, temperature) or chemical (e.g., nutrients, pH, oxygen) properties of the environment, has the potential to increase species turnover. Consequently, disturbance is either a useful tool to displace unwanted residents [46]; see Biotic Interactions) or a barrier that impedes inoculant establishment. In highly disturbed environments, long-term establishment may be an unrealistic goal and persistent delivery of inoculants may be unavoidable. For example, in phyllospheres, soils, or rivers, ‘washouts’ of microbes may occur from heavy or frequent precipitation events or other physical disruptions [47]. Periodic chemical disturbances, such bile secretion in the gut [48] or application of pesticides in soils [49] can also impact inoculant establishment. Providing inoculants with an enduring physical haven that allows continuous dispersal into surrounding space may overcome disturbance barriers, as seen in some macrobial and microbial systems [16, 50]. This strategy is enhanced when microbial inoculants possess superior abilities for attachment [51], biofilm formation [52], or stress resistance [53].

### Potential barrier: biotic interactions

The variety of BI is complex but can be broadly categorized as either antagonistic or facilitative [54]. Within these categories, interactions can be described as direct or indirect in relation to an organism of interest. For example, antagonistic direct interactions include mechanisms such as predation or competition, while antagonistic indirect interactions include environmental modifications like antibiotic production or pH changes may adversely affect competitors and non-competitors alike. In macrobial ecology, the impacts of direct effects have been more commonly considered e.g., [55], but research on indirect effects is increasing e.g., [56]. There is also growing recognition that the number or complexity of interactions may affect establishment in macrobial [57] and microbial systems [58]. In simple systems with cultured microbes, antagonistic interactions are abundant and are a driving force in community assembly and stability [59]. Antagonism also plays a role in functioning of more complex microbial systems [60] and may be used in microbiome engineering [61]. Like antagonism, facilitative interactions can also be direct or indirect. For example, the presence of a keystone species that provides a resource supporting an inoculant is direct facilitation, whereas a general modification of the environment (e.g., a change in soil pH) that benefits an inoculant is indirect facilitation. The distinction between direct and indirect BI is important for microbiome engineering because preoccupation with direct interactions can overlook important indirect barriers and solutions.

#### Antagonism via simple competition

The complexity of competition can be categorized by the mechanisms organisms use to capture growth limiting resources. In the simple case, competition involves differences in search capability (i.e., motility) and/or resource capture efficiency (e.g., nutrient uptake via transporters). Increasing the number of competitors for a growth-limiting substrate may reduce the ability of an inoculant to capture a sufficient quota of resource to survive [62]. A general trend observed in multiple ecosystems is that increased resident community diversity generally reduces invasibility because diverse communities leave less free niche space [63, 64]. For microbiome engineering solutions to overcome a competition barrier might include creating a protected physical space or adding resources to support the inoculant during an establishment period [58, 65].

#### Antagonism via antibiotics

Complex competition involves additional strategies to undermine competitors. Strategies include secretion of antibiotics, signaling compounds that adversely affect the metabolism of other species (e.g., quorum signals or volatile organic compounds), or siderophores that create new growth limitations for competitors. Antibiotic production is the best studied strategy. In pairwise interactions of cultured isolates, antagonistic antibiotic production occurred in approximately half of *Bacillus* [66] and *Streptomyces* [67] isolates. Antagonistic growth inhibition tends to increase with phylogenetic similarity of species [68], presumably because competition is greatest among close relatives with similar traits, a concept shared across microbial and macrobial biology. The impact of antibiotics in natural communities is illustrated by the production of andrimid by marine bacteria, which inhibits *Vibrio cholerae* growth [69]. The extent to which antibiotic production is a barrier to establishment of inoculants in microbiome engineering remains to be established, but has been shown in at least one instance [70] and might be routinely assayed by inhibition assays with filtrates from a resident community. Active antagonism may also be an engineering tool for establishment, illustrated by an artificial biocontrol strain used to remove unwanted bacteria in a community [71].

#### Antagonism via predation

Another type of antagonistic interaction that may influence the ability of inoculants to establish is predation. Predation by protists, bacteria, and fungi is likely to be density dependent, not taxon-specific, although cases of the latter have been documented [61]. Predation by host-specific viruses may also impact invader success. Phages vary in host range but generally display host specificity. While phage are known to impact the diversity and function of microbial communities [72], they are still rarely considered when assessing controls on microbiome succession [73]. Phages may stabilize the co-existence of competing bacteria by preventing dominance of a single species—an example of the “kill-the-winner” hypothesis [74]. In microbiome engineering, establishing inoculants with “naïve immunity” may fail owing to lysis by phages in the resident community [75]. Conversely, predation can be a tool for tailored removal of residents that impede establishment of desired strains, by building on concepts from work using phages for biocontrol of pathogens in humans, on foods, in aquaculture, and on plants (e.g., [76]).

#### Facilitation

Stable facilitative interactions between microorganisms are only expected in restricted cases [77]. For example, environmental conditions can mediate a tradeoff between facilitation and competition, where harsher environments foster microbial facilitation [78]. Another view posits that microbial communities are able to organize into metabolically cohesive units where consortia with positive feedback loops use resources in a stable manner and minimize competition through resource specialization and exclusion of resource generalists [79]. For microbiome engineering, facilitative interactions could be leveraged, for example by creating highly specialized microbial units where organisms are filling different niches.

### Summary

**Fig. 1:**
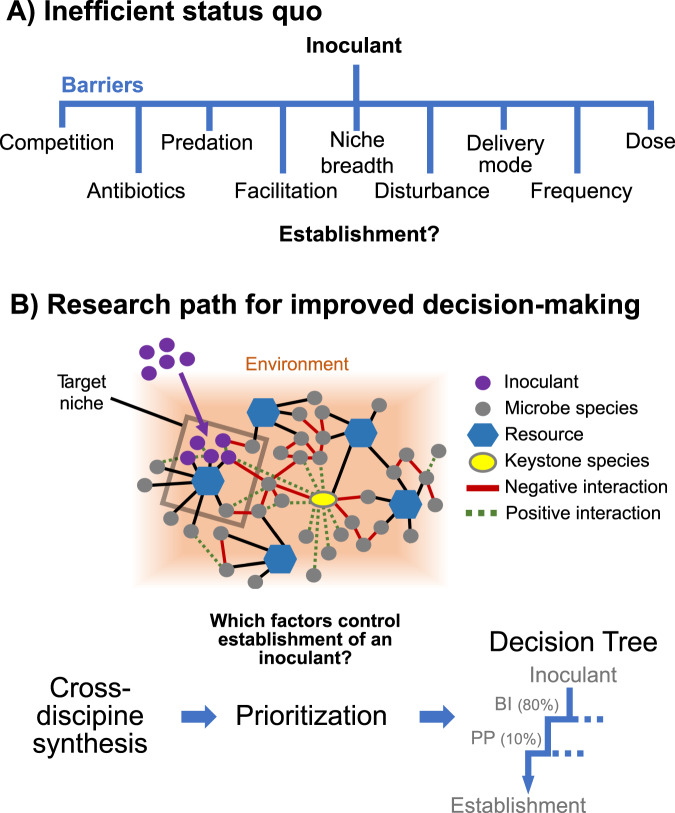
Barriers to organism establishment and a path forward for microbiome engineering. **A** The current state of research focused on inoculum establishment. Without quantitative prioritization, potential barriers like the illustrated examples are investigated randomly. **B** Path forward to improve establishment success. Prioritization of barriers includes quantifying their relative impact in order to create decision trees that can simultaneously rank barriers and summarize the potential impact of overcoming each barrier in a multi-factor solution path. Values in parentheses on the decision tree illustrate an example of percentage impact of a barrier on inoculum establishment, guiding microbiome engineering investments.

The framework of PP, EF, and BI is a simple way of categorizing a long list of ecological phenomena that can be barriers (or engineering tools) for establishment of inocula in resident communities (Table 1, Table S1). The overview above highlights a few of the many sub-factors that have been documented in macrobial ecology [13]. Given the large number and context-dependence of factors that may impede organism establishment, ranking barriers at the highest level (PP, EF, and BI) in a decision tree framework is a critical first step to accelerate microbiome engineering and avoid random testing of factors (Fig. 1).

Decision trees are increasingly being used in macrobial ecology to manage species invasions and ecological restoration. For example, some decision trees prioritize actions based on the potential severity of invasive species on ecosystem services [80]. In a restoration ecology examples, decision trees based on seed dormancy attributes [81] or optimal habitat characteristics for bird nesting [82] can guide restoration plans. Many decision trees available to managers in macrobial ecology include a decision-making step related to establishment (e.g., is there adequate time for a seedling to establish during its typical growing season?).

Building decision trees in microbiome engineering requires studies that test the relative importance of factors on establishment success. For example, a recent study found that the relative importance of PP and BI varied by organism domain, bacteria versus fungi [19]. Another study found that the impacts of PP depended on the competitive interactions of residents and inoculants [18]. Findings from many studies like these must be averaged to elucidate *general* rules for decision trees, but such a pursuit is inefficient without planning for quantitative synthesis.

## Breaking barriers: synthesis and recommendations

Overall, we posit that increasing focus on BI will lead to increased success in inoculant establishment and improve outcomes in microbiome engineering (Fig. 1B). While BI are commonly considered in invasion biology and restoration ecology, manipulating BI is an under-explored mechanism in microbiome engineering, likely due to the abundance and complexity of organism interactions in any given microbiome. Greater success in manipulating early succession ecosystems (e.g., infant compared to adult guts) [38, 65] may be due in part to their lower complexity, which would point to the importance of BI. Assessing the number and type of BI relevant for engineering complex microbiomes is challenging because of the lack of direct measurement capabilities. Network reconstruction techniques based on correlated species abundances are often used to infer interactions (e.g., [83]), but further development is needed to move beyond speculative inferences [84]. Manipulation of microbial keystone species deserves attention as a potential solution in microbiome engineering as this concept has proven to be a powerful strategy in restoration ecology [85]. Although keystone microbial taxa have been predicted in co-occurrence networks [86] and linked to compositional shifts [87], few studies have confirmed the physiological role of keystone microbes in a community [88].

In order to confirm the role of BI in microbiome engineering outcomes and refine a decision tree of barriers to organism establishment we recommend a number of actions. First, greater attention is needed in microbial research to measure establishment (persistence and/or proliferation) of inocula over long timescales (e.g., months to years) with whole-community measurement techniques that offer broader insight and more standardized experimental design and reporting to facilitate meta-analyses. Second, a careful cross-discipline synthesis—i.e., meta-analyses of microbial studies that measure the dependence of inocula establishment on aspects of BI, as well as PP and EF, that would illuminate knowledge gaps is needed. We expand on these concepts in the sections below.

### Recommendations for future research

To accelerate microbiome engineering across different applications, there is a need to target knowledge gaps that are contingent on experimental design. Gaps range from complexity of the inoculum to multifactorial manipulation strategies. We highlight four gaps. (1) To date, most microbial establishment studies have one or a few inoculant species. In contrast, transplant of entire microbiomes (i.e., fecal transplants and activated sludge transplants) is an increasing practice for some medical and wastewater treatment applications, respectively, yet knowledge of how these complex invasions impact community composition and functioning is limited. (2) Often microbiome engineering studies measure functional changes, not inoculant establishment. Monitoring inoculants is needed to determine if failure to achieve desired functional changes is due to lack of establishment or instead, to attenuation of desired functions in established inoculants. (3) More insight into the temporal dynamics of functional changes is needed. It is common practice to assess function at only one or two timepoints. Furthermore, the time interval between manipulation and functional assessment varies from hours to weeks. In many studies functional measurements occur immediately following probiotic intervention and longer-term impacts are not assessed, limiting insights into barriers to establishment. (4) It is likely that multiple factors simultaneously contribute to the failure or success of species establishment (e.g., see [18]). Thus, multifactorial experiments that test the interactive effects of establishment barriers (or engineering solutions) are needed and may be especially helpful in building decision trees.

### Translating among fields

Prioritizing establishment barriers through cross-discipline synthesis depends on the capacity to find related knowledge that is obscured by discipline-specific jargon. For example, medical research on microbiome engineering typically uses the term “probiotics” [89], while agricultural studies use the terms “microbial inoculants”, “plant growth promoting microbes” (PGPM’s), “biocontrol agents”, plant “biostimulants” or “probiotics” [90]. To facilitate cross-discipline synthesis, we compiled some key concepts, terms, and definitions (Table S2)—a step towards making related knowledge findable, accessible, interoperable, and reusable (FAIR), as recommended by the National Microbiome Data Collaborative (NMDC; [91]).

### Experimental design

Some standardization of experimental design features (e.g., independent and dependent variables, replication, testing scale, and temporal sampling) can also facilitate synthesis. At present, wide variation in experimental design [92] impedes this goal. For example, functional changes in the gut microbiome might be assessed in some studies qualitatively from statements of symptom relief among patients [93], whereas other studies may quantify changes in the concentration of a specific analyte [94]. Microbial community composition is often reported in a qualitative way; whereas variance partitioning would facilitate quantification of probiotic impacts among studies. Furthermore, use of positive controls (mock communities) and/or internal standards in amplicon sequencing for taxonomic profiling can improve the quantitative insights of this common and relatively low-cost measurement technique [95]. Including a link in publications to an easily accessible data table summarizing experimental design can also facilitate data syntheses within and across disciplines. A database of searchable studies that include the experimental design and results with permanent identification records and rich contextual metadata (somewhat analogous to clinicalstudies.info.nih.gov/) would greatly accelerate progress. Publicly accessible genomic repositories are a prime example of how data aggregation and standardized formatting across disparate studies is leading to groundbreaking discoveries [96]. Data attribution guidelines that give researchers credit for datasets used in subsequent work [96, 97] is another useful step.

## Conclusions

Microbiome engineering is a rapidly expanding field. There are notable cases of success in microbiome engineering for human health, in particular in the infant gut [38, 65], bioremediation [98], wastewater engineering [99], and agriculture [100]. However, inoculants often fail to establish or to modify ecosystem functioning over significant time periods [8]. Dispersal, environmental, and biotic barriers to organism establishment likely contribute to failures. Given the complex suite of possible barriers, developing a decision tree to prioritize barriers is a top priority to guide engineering. This priority may be aided by cross-disciplinary synthesis because disparate fields are tackling similar challenges. However, to leverage research and unite findings across fields there is a need to recognize differences in terminology and to standardize reporting of tested factors and magnitude of effects. Lastly, increased attention to the types of experiments performed and extended time-course measurements of inoculant establishment will provide insights that accelerate successful microbiome engineering across a range of applications.

## Supplementary information

Table S1

Table S2
