# Supplementary material for: Solutions in microbiome engineering: prioritizing barriers to organism establishment
Source: ISME J. 2021 Aug 21;16(2):331–8. doi: 10.1038/s41396-021-01088-5 (PMC8776856; doi:10.1038/s41396-021-01088-5)
Supplement: Supplementary file 1 — Table S1 [file 41396_2021_1088_MOESM1_ESM.docx]

**Table S1.** Some examples of relevant literature across fields pertaining to establishment barriers and solutions.

| **Ecological Principle** | **Field** | **Relevant Literature** |
| --- | --- | --- |
| **Propagule Pressure (PP)** |  |  |
| Dose/Frequency | Invasion biology | Catford et al. 2009, Colautti et al. 2006, Lockwood et al. 2005, Simberloff et al. 2009, Von Holle and Simberloff 2005, Wittmann et al. 2014 |
|  | Restoration ecology | Corbin and Holl 2012, Funk et al. 2012, Hulvey et al. 2017, Schantz et al. 2015, Wainwright et al. 2018 |
|  | Microbial ecology | Acosta et al 2015, Albright et al. 2020, De Roy et al. 2013, Fodelianakis et al 2020, Jones et al. 2017, Ketola et al. 2017,Mallon et al. 2015, Rivett et al. 2018, van Elsas et al. 2012, Vila et al. 2019, Yang et al. 2017, Zhou and Ning 2017 |
|  | Human/animal microbiome engineering | Khan and Chousalkar 2020, Nguyen et al 2018, Petschow et al. 2005, Xing et al. 2017 |
|  | Agriculture/soil microbiome engineering | Bai et al. 2002 |
|  | Wastewater / Drinking water treatment |  |
|  | Bioremediation | Comeau et al. 1993, Nam et al. 2005 |
|  | Microbiome engineering (other) | \| Choudhary and Schmidt-Dannert 2010 \| \| --- \| |
| Delivery Mode | Invasion biology | Kreitschitz et al. 2021 |
|  | Restoration ecology | Gornish et al. 2019 |
|  | Microbial ecology |  |
|  | Human/animal microbiome engineering |  |
|  | Agriculture/soil microbiome engineering | Howard et al. 2017, Kaminsky et al. 2019, O'Callaghan 2016 |
|  | Wastewater / Drinking water treatment | Ali et al. 2015 |
|  | Bioremediation | Power et al. 2011 |
|  | Microbiome engineering (other) | Du et al. 2019 |
| **Environmental Filtering (EF)** |  |  |
| Niche availability | Invasion biology | Cadotte et al. 2018, Gallien et al. 2015, Maitner et al. 2012 |
|  | Restoration ecology | Fick et al. 2016, Fick et al. 2020, James et al. 2019, Nuttle et al 2007, Sheley et al. 2009, Vasquez et al. 2011, Wainwright et al. 2018 |
|  | Microbial ecology | Bell and Bell 2021, Fahimipour and Gross 2020, Muthukrishnan et al. 2018, Sriswadsi et al. 2017, Zampieri et al. 2019, Zhao et al. 2019 |
|  | Human/animal microbiome engineering | Campieri et al. 2000, Costabile et al. 2017, Enam and Mansell 2019, Freter et al. 1983, Frese et al. 2017, Markowiak and Slizewska 2017, McNally and Brown 2015, Pereira and Berry 2017, Perraudeau et al. 2020, Pokusaeva et al. 2011, Shepard et al. 2018, Umu et al. 2017 |
|  | Agriculture/soil microbiome engineering | Arif et al. 2020, Albareda et al. 2008, Haskett et al. 2020, Lennon et al. 2012, Remans et al. 2007, Sasse et al. 2018 |
|  | Wastewater / Drinking water treatment |  |
|  | Bioremediation | Loffler and Edwards 2006, Otte et al. 1994, Thompson et al. 2005 |
|  | Microbiome engineering (other) | Shahab et al. 2020, Shaw et al. 2016 |
| Disturbance | Invasion biology | Lembrechts et al. 2016, Miller et al. 2021 |
|  | Restoration ecology | Corbin and Holl 2012, Hulvey et al. 2017 |
|  | Microbial ecology | Gibbons et al. 2016, Shade et al. 2012 |
|  | Human/animal microbiome engineering | Ali et al. 2020, Bezkorovainy 2001, Lewis et al. 2018, Praveschotinunt et al 2019, Sharma and Kanwar 2017, Zhao et al. 2020 |
|  | Agriculture/soil microbiome engineering | Bach and Hofmockel 2016, Beauregard et al. 2013, de Souza et al. 2015, Maltz and Treseder 2015, Tripathi et al. 2020, Upton et al. 2019 |
|  | Wastewater / Drinking water treatment | Mansfeldt et al. 2019, Zhang et al. 2018, Wang et al. 2020 |
|  | Bioremediation |  |
|  | Microbiome engineering (other) | Baltzis and Frederickson 1983, Dykhuizen and Hartl 1983, Lewis et al. 2018 |
| **Biotic interactions (BI)** |  |  |
| Antagonism Via Simple Competition | Invasion biology | Beaury et al. 2020, Corbin and D'Antonio et al. 2004 |
|  | Restoration ecology | Herron et al. 2013 |
|  | Microbial ecology | Bauer et al. 2018, Eisenhauer et al. 2013, Fuschlin et al. 2012, Hibbing et al. 2010 |
|  | Human/animal microbiome engineering | Panigrahi et al. 2018, Perraudeau et al. 2020, Suez et al. 2018 |
|  | Agriculture/soil microbiome engineering | Capdevilla et al. 2004 |
|  | Wastewater / Drinking water treatment |  |
|  | Bioremediation |  |
|  | Microbiome engineering (other) |  |
| Antagonism Via Antibiotics | Invasion biology |  |
|  | Restoration ecology |  |
|  | Microbial ecology | Long et al. 2005, Perrez-Gutierrez et al. 2013, Russel et al. 2017, Safferman and Morris 1962 |
|  | Human/animal microbiome engineering | Hecht et al. 2016 |
|  | Agriculture/soil microbiome engineering | Deoliveira et al. 1995 |
|  | Wastewater / Drinking water treatment |  |
|  | Bioremediation |  |
|  | Microbiome engineering (other) | Dwidar et al. 2015 |
| Antagonism Via Predation | Invasion biology | Blumenthal 2005, Keane and Crawley 2002, Kinnear et al. 2002, Mitchell and Power 2003 |
|  | Restoration ecology |  |
|  | Microbial ecology | Bahr 1954, Barker and Brown 1994, Greub and Raoult 2004, Koskella 2019, Matz and Kjelleberg 2005, Pernthaler et al. 2005, Shapiro et al. 2010, Soundararajan et al. 2019, Thingstad 2000 |
|  | Human/animal microbiome engineering | Das et al. 2015, Dedrick et al. 2019, Nale et al. 2018, Ott et al. 2017 |
|  | Agriculture/soil microbiome engineering | Balogh et al. 2010, Marsh and Wellington 1994, Postma et al. 1990, Wang et al. 2019, Wright et al. 1995 |
|  | Wastewater / Drinking water treatment | Feitchmayer et al. 2017, Manefield et al 2007 |
|  | Bioremediation | Bouchez et al. 2000 |
|  | Microbiome engineering (other) | Barrangou et al. 2007, Lopez-Igual et al. 2019, Nakai and Park 2002 |
| Facilitation | Invasion biology |  |
|  | Restoration ecology | Byers et al 2006, Gagnon et al. 2020, Valdez et al. 2020 |
|  | Microbial ecology | Foster and Bell 2012, Pascual-Garcia et al. 2020 |
|  | Human/animal microbiome engineering |  |
|  | Agriculture/soil microbiome engineering | Anandham et al. 2007 |
|  | Wastewater / Drinking water treatment |  |
|  | Bioremediation | Piccardi et al. 2019 |
|  | Microbiome engineering (other) | Li et al. 2017, Zhang et al. 2020 |
|  |  |  |
| Not Categorized | Invasion biology | Berner and Bruckhart 2005, Corbin and D'Antonio et al. 2004, Gallien and Carboni 2017, Kinnear et al. 2002, Mitchell et al. 2006, Northfield et al. 2018, Seastedt and Pysek 2011, Tillman et al. 2004, White et al. 2006 |
|  | Restoration ecology | Carroll 2017, Estes et al. 2011, Funk et al. 2008, Paine et al. 1966, Palmer et al. 1997, Ripple and Beschta 2012, Ripple et al. 2014, Shea and Chesson 2002, Sinclair et al. 2007 |
|  | Microbial ecology | Albright et al. 2020, Berry and Widder 2014, Brunel et al. 2020, Garcia-Bayona and Comstock 2018, Herren and McMahon 2018, Maynard et al. 2017, Zapien-Campos et al. 2015 |
|  | Human/animal microbiome engineering | Baktash et al. 2018, Frese et al. 2017, Panigrahi et al. 2018, Seekatz et al. 2014, Suez et al. 2018, Trosvik and de Muinck 2015 |
|  | Agriculture/soil microbiome engineering |  |
|  | Wastewater / Drinking water treatment |  |
|  | Bioremediation |  |
|  | Microbiome engineering (other) | Kong et al. 2018 |
|  |  |  |

**REFERENCES:**

|  |
| --- |
| Acosta F, Zamor RM, Najar FZ, Roe BA, Hambright KD. Dynamics of an experimental microbial invasion. Proc Natl Acad Sci USA. 2015;112(37):11594-9. |
| Albareda M, Rodriguez-Navarro DN, Camacho M, Temprano FJ. Alternatives to peat as a carrier for rhizobia inoculants: Solid and liquid formulations. Soil Biol Biochem. 2008;40(11):2771-9. |
| Albright MBN, Sevanto S, Gallegos Graves LV, Dunbar J. Biotic interactions are more important than propagule pressure in microbial community invasions. Mbio. 2020;11:e02089-20. |
| Ali M, Oshiki M, Rathnayake L, Ishii S, Satoh H, Okabe S. Rapid and successful start-up of anammox process by immobilizing the minimal quantity of biomass in PVA-SA gel beads. Water Res. 2015;79:147-57. |
| Ali SA, Singh P, Tomar SK, Mohanty AK, Behare P. Proteomics fingerprints of systemic mechanisms of adaptation to bile in Lactobacillus fermentum. J Proteom. 2020;213:103600. |
| Anandham R, Sridar R, Nalayini P, Poonguzhali S, Madhaiyan M, Sa T. Potential for plant growth promotion in groundnut (Arachis hypogaea L.) cv. ALR-2 by co-inoculation of sulfur-oxidizing bacteria and Rhizobium. Microbiol Res. 2007;162(2):139-53. |
| Arif I, Batool M, Schenk PM. Plant Microbiome Engineering: Expected benefits for improved crop growth and resilience. Trends Biotechnol. 2020;38(12):1385-1396. |
| Bach EM, Hofmockel KS. A time for every season: soil aggregate turnover stimulates decomposition and reduces carbon loss in grasslands managed for bioenergy. Glob Change Biol Bioenergy. 2016;8(3):588-99. |
| Bahr H. *Untersuchungen uber die rolle der ciliaten als bakterienvernichter im rahmen der biologischen reinigung des abwassers. Z Hyg Infektionskr. 1954;139(2):160-81. |
| Bai YM, Pan B, Charles TC, Smith DL. Co-inoculation dose and root zone temperature for plant growth promoting rhizobacteria on soybean [Glycine max (L.) Merr] grown in soil-less media. Soil Biol Biochem. 2002;34(12):1953-7. |
| Baktash A, Terveer EM, Zwittink RD, Hornung BVH, Corver J, Kuijper EJ, et al. Mechanistic insights in the success of fecal microbiota transplants for the treatment of clostridium difficile infections. Front Microbiol. 2018;9:1242. |
| Balogh B, Jones JB, Iriarte FB, Momol MT. Phage therapy for plant disease control. Curr Pharm Biotechnol. 2010;11(1):48-57. |
| Baltzis BC, Fredrickson AG. Competition of two microbial populations for a single resource in a chemostat when one of them exhibits wall attachment. Biotechnol Bioeng. 1983;25(10):2419-39. |
| Barker J, Brown MRW. Trojan-Horses of the Microbial World - Protozoa and the survival of bacterial pathogens in the environment. Br Microbiol Res J. 1994;140:1253-9. |
| Barrangou R, Fremaux C, Deveau H, Richards M, Boyaval P, Moineau S, et al. CRISPR provides acquired resistance against viruses in prokaryotes. Science. 2007;315(5819):1709-12. |
| Bauer MA, Kainz K, Carmona-Gutierrez D, Madeo F. Microbial wars: Competition in ecological niches and within the microbiome. Microbiol Cell. 2018;5(5):215-9. |
| Beauregard PB, Chai YR, Vlamakis H, Losick R, Kolter R. Bacillus subtilis biofilm induction by plant polysaccharides. Proc Natl Acad Sci USA. 2013;110(17):E1621-E30. |
| Beaury EM, Finn JT, Corbin JD, Barr V, Bradley BA. Biotic resistance to invasion is ubiquitous across ecosystems of the United States. Ecol Lett. 2020;23(3):476-82. |
| Bell TH, Bell T. Many roads to bacterial generalism. Fems Microbiol Ecol. 2021;97(1):fiaa240 |
| Berner DK, Bruckart WL. A decision tree for evaluation of exotic plant pathogens for classical biological control of introduced invasive weeds. Biol Control. 2005;34(2):222-32. |
| Berry D, Widder S. Deciphering microbial interactions and detecting keystone species with co-occurrence networks. Front Microbiol. 2014;5:219. |
| Bezkorovainy A. Probiotics: determinants of survival and growth in the gut. Am J Clin Nutr. 2001;73(2):399s-405s. |
| Blumenthal D. Ecology - Interrelated causes of plant invasion. Science. 2005;310(5746):243-4. |
| Bouchez T, Patureau D, Dabert P, Juretschko S, Dore J, Delgenes P, et al. Ecological study of a bioaugmentation failure. Environ Microbiol. 2000;2(2):179-90. |
| Brunel C, Pouteau R, Dawson W, Pester M, Ramirez KS, van Kleunen M. Towards unraveling macroecological patterns in rhizosphere microbiomes. Trends Plant Sci. 2020;25(10):1017-29. |
| Byers JE, Cuddington K, Jones CG, Talley TS, Hastings A, Lambrinos JG, et al. Using ecosystem engineers to restore ecological systems. Trends Ecol Evol. 2006;21(9):493-500. |
| Cadotte MW, Campbell SE, Li SP, Sodhi DS, Mandrak NE. Preadaptation and naturalization of nonnative species: Darwin's two fundamental insights into species invasion. Annu Rev Plant Biol. 2018;69:661-84. |
| Campieri M, Rizzello F, Venturi A, Poggioli G, Ugolini F, Helwig U, et al. Combination of antibiotic and probiotic treatment is efficacious in prophylaxis of post-operative recurrence of Crohn's disease: A randomized controlled study vs mesalamine. Gastroenterology. 2000;118(4):A781-A. |
| Capdevila S, Martinez-Granero FM, Sanchez-Contreras M, Rivilla R, Martin M. Analysis of Pseudomonas fluorescens F113 genes implicated in flagellar filament synthesis and their role in competitive root colonization. Microbiol-SGM. 2004;150:3889-97. |
| Carroll SB. The Serengeti rules: The quest to discover how life works and why it matters: Princeton University Press; 2017. |
| Catford JA, Jansson R, Nilsson C. Reducing redundancy in invasion ecology by integrating hypotheses into a single theoretical framework. Divers Distrib. 2009;15(1):22-40. |
| Choudhary S, Schmidt-Dannert C. Applications of quorum sensing in biotechnology. Appl Microbiol Biotechnol. 2010;86(5):1267-79. |
| Colautti RI, Grigorovich IA, MacIsaac HJ. Propagule pressure: A null model for biological invasions. Biol. Invasions. 2006;8(5):1023-37. |
| Comeau Y, Greer CW, Samson R. Role of inoculum preparation and density on the bioremediation of 2,4-D-contaminated soil by bioaugmentation. Appl Microbiol Biotechnol. 1993;38(5):681-7. |
| Corbin JD, D'Antonio CM. Competition between native perennial and exotic annual grasses: Implications for an historical invasion. Ecology. 2004;85(5):1273-83. |
| Corbin JD, Holl KD. Applied nucleation as a forest restoration strategy. For Ecol Manag. 2012;265:37-46. |
| Costabile A, Bergillos-Meca T, Rasinkangas P, Korpela K, de Vos WM, Gibson GR. Effects of soluble corn fiber alone or in synbiotic combination with Lactobacillus rhamnosus GG and the pilus-deficient derivative GG-PB12 on fecal microbiota, metabolism, and markers of immune function: a randomized, double-blind, placebo-controlled, crossover study in healthy elderly (Saimes study). Front Immunol. 2017;8:1443. |
| Das M, Bhowmick TS, Ahern SJ, Young R, Gonzalez CF. Control of Pierce's disease by phage. PloS One. 2015;10(6):e0128902. |
| De Roy K, Marzorati M, Negroni A, Thas O, Balloi A, Fava F, et al. Environmental conditions and community evenness determine the outcome of biological invasion. Nat Commun. 2013;4:1383. |
| de Souza R, Ambrosini A, Passaglia LMP. Plant growth-promoting bacteria as inoculants in agricultural soils. Genet Mol Biol. 2015;38(4):401-19. |
| Dedrick RM, Guerrero-Bustamante CA, Garlena RA, Russell DA, Ford K, Harris K, et al. Engineered bacteriophages for treatment of a patient with a disseminated drug-resistant Mycobacterium abscessus. Nat Med. 2019;25(5):730-733. |
| Deoliveira RDB, Wolters AC, Vanelsas JD. Effects of antibiotics in soil on the population-dynamics of transposon Tn5 carrying Pseudomonas-fluorescens. Plant Soil. 1995;175(2):323-33. |
| Du Y, Zhou SH, Liu M, Wang BJ, Jiang KY, Fang H, et al. Understanding the roles of surface proteins in regulation of Lactobacillus pentosus HC-2 to immune response and bacterial diversity in midgut of Litopenaeus vannamei. Fish Shellfish Immunol. 2019;86:1194-206. |
| Dwidar M, Nam D, Mitchell RJ. Indole negatively impacts predation by Bdellovibrio bacteriovorus and its release from the bdelloplast. Environ Microbiol. 2015;17(4):1009-22. |
| Dykhuizen DE, Hartl DL. Selection in chemostats. Microbiol Rev. 1983;47(2):150-68. |
| Eisenhauer N, Schulz W, Scheu S, Jousset A. Niche dimensionality links biodiversity and invasibility of microbial communities. Funct Ecol. 2013;27(1):282-8. |
| Enam F, Mansell TJ. Prebiotics: tools to manipulate the gut microbiome and metabolome. J Ind Microbiol Biotechol. 2019;46(9-10):1445-59. |
| Estes JA, Terborgh J, Brashares JS, Power ME, Berger J, Bond WJ, et al. Trophic downgrading of planet Earth. Science. 2011;333(6040):301-6. |
| Fahimipour AK, Gross T. Mapping the bacterial metabolic niche space. Nat Commun. 2020;11(1):4887. |
| Feichtmayer J, Deng L, Griebler C. Antagonistic Microbial Interactions: Contributions and potential applications for controlling pathogens in the aquatic systems. Front Microbiol. 2017;8:2192. |
| Fick SE, Day N, Duniway MC, Hoy-Skubik S, Barger NN. Microsite enhancements for soil stabilization and rapid biocrust colonization in degraded drylands. Restor Ecol. 2020;28:S139-S49. |
| Fick SE, Decker C, Duniway MC, Miller ME. Small-scale barriers mitigate desertification processes and enhance plant recruitment in a degraded semiarid grassland. Ecosphere. 2016;7(6):e01354. |
| Fodelianakis S, Valenzuela-Cuevas A, Barozzi A, Daffonchio D. Direct quantification of ecological drift at the population level in synthetic bacterial communities. ISME J. 2021;15:55-66. |
| Foster KR, Bell T. Competition, not cooperation, dominates interactions among culturable microbial species. Curr Biol. 2012;22(19):1845-50. |
| Frese SA, Hutton AA, Contreras LN, Shaw CA, Palumbo MC, Casaburi G, et al. Persistence of supplemented Bifidobacterium longum subsp. infantis EVC001 in breastfed infants. Msphere. 2017;2(6). |
| Freter R, Brickner H, Botney M, Cleven D, Aranki A. Mechanisms that control bacterial-populations in continuous-flow culture models of mouse large intestinal flora. Infect Immunol. 1983;39(2):676-85. |
| Fuchslin HP, Schneider C, Egli T. In glucose-limited continuous culture the minimum substrate concentration for growth, s(min), is crucial in the competition between the enterobacterium Escherichia coli and Chelatobacter heintzii, an environmentally abundant bacterium. ISME J. 2012;6(4):777-89. |
| Funk JL, Cleland EE, Suding KN, Zavaleta ES. Restoration through reassembly: plant traits and invasion resistance. Trends Ecol Evol. 2008;23(12):695-703. |
| Funk JL, Hoffacker MK, Matzek V. Summer irrigation, grazing and seed addition differentially influence community composition in an invaded serpentine grassland. Restor Ecol. 2015;23(2):122-30. |
| Gagnon K, Rinde E, Bengil EGT, Carugati L, Christianen MJA, Danovaro R, et al. Facilitating foundation species: The potential for plant-bivalve interactions to improve habitat restoration success. J Appl Ecol. 2020;57(6):1161-79. |
| Gallien L, Carboni M. The community ecology of invasive species: where are we and what's next? Ecography. 2017;40(2):335-352. |
| Gallien L, Mazel F, Lavergne S, Renaud J, Douzet R, Thuiller W. Contrasting the effects of environment, dispersal and biotic interactions to explain the distribution of invasive plants in alpine communities. Biol Invasions. 2015;17(5):1407-23. |
| Garcia-Bayona L, Comstock LE. Bacterial antagonism in host-associated microbial communities. Science. 2018;361(6408):eeat2456. |
| Gibbons SM, Scholz M, Hutchison AL, Dinner AR, Gilbert JA, Coleman ML. Disturbance regimes predictably alter diversity in an ecologically complex bacterial system. Mbio. 2016;7(6):e01372-16. |
| Gornish E, Arnold H, Fehmi J. Review of seed pelletizing strategies for arid land restoration. Restor Ecol. 2019;27(6):1206-11. |
| Greub G, Raoult D. Microorganisms resistant to free-living amoebae. Clin Microbiol Rev. 2004;17(2):413-33. |
| Haskett TL, Tkacz A, Poole PS. Engineering rhizobacteria for sustainable agriculture. ISME J. 2021;15:949-964. |
| Hecht AL, Casterline BW, Earley ZM, Goo YA, Goodlett DR, Wardenburg JB. Strain competition restricts colonization of an enteric pathogen and prevents colitis. EMBO Rep. 2016;17(9):1281-91. |
| Herren CM, McMahon KD. Keystone taxa predict compositional change in microbial communities. Environ Microbiol. 2018;20(6):2207-17. |
| Herron CM, Jonas JL, Meiman PJ, Paschke MW. Using native annual plants to restore post-fire habitats in western North America. Int J Wildland Fire. 2013;22(6):815-21. |
| Hibbing ME, Fuqua C, Parsek MR, Peterson SB. Bacterial competition: surviving and thriving in the microbial jungle. Nat Rev Microbiol. 2010;8(1):15-25. |
| Howard MM, Bell TH, Kao-Kniffin J. Soil microbiome transfer method affects microbiome composition, including dominant microorganisms, in a novel environment. FEMS Microbiol Lett. 2017;364(11):fnx092. |
| Hulvey KB, Leger EA, Porensky LM, Roche LM, Veblen KE, Fund A, et al. Restoration islands: a tool for efficiently restoring dryland ecosystems? Restor Ecol. 2017;25:S124-S34. |
| James JJ, Sheley RL, Leger EA, Adler PB, Hardegree SP, Gornish ES, et al. Increased soil temperature and decreased precipitation during early life stages constrain grass seedling recruitment in cold desert restoration. J Appl Ecol. 2019;56(12):2609-19. |
| Jones ML, Ramoneda J, Rivett DW, Bell T. Biotic resistance shapes the influence of propagule pressure on invasion success in bacterial communities. Ecology. 2017;98(7):1743-9. |
| Kaminsky LM, Trexler RV, Malik RJ, Hockett KL, Bell TH. The inherent conflicts in developing soil microbial inoculants. Trends Biotechnol. 2019;37(2):140-51. |
| Keane RM, Crawley MJ. Exotic plant invasions and the enemy release hypothesis. Trends Ecol Evol. 2002;17(4):164-70. |
| Ketola T, Saarinen K, Lindstrom L. Propagule pressure increase and phylogenetic diversity decrease community's susceptibility to invasion. BMC Ecol. 2017;17:15. |
| Khan S, Chousalkar KK. Salmonella Typhimurium infection disrupts but continuous feeding of Bacillus based probiotic restores gut microbiota in infected hens. J Anim Sci Biotechnol. 2020;11(1):29. |
| Kinnear JE, Sumner NR, Onus ML. The red fox in Australia - an exotic predator turned biocontrol agent. Biol Conserv. 2002;108(3):335-59. |
| Kong WT, Meldgin DR, Collins JJ, Lu T. Designing microbial consortia with defined social interactions. Nat Chem Biol. 2018;14(8):821-29. |
| Koskella B. New approaches to characterizing bacteria-phage interactions in microbial communities and microbiomes. Env Microbiol Rep. 2019;11(1):15-6. |
| Kreitschitz A, Haase E, Gorb SN. The role of mucilage envelope in the endozoochory of selected plant taxa. Sci Nat-Heidelberg. 2021;108(1):2. |
| Lembrechts JJ, Pauchard A, Lenoir J, Nunez MA, Geron C, Ven A, et al. Disturbance is the key to plant invasions in cold environments. Proc Natl Acad Sci USA. 2016;113(49):14061-6. |
| Lennon JT, Aanderud ZT, Lehmkuhl BK, Schoolmaster DR. Mapping the niche space of soil microorganisms using taxonomy and traits. Ecology. 2012;93(8):1867-79. |
| Lewis DD, Vanella R, Vo C, Rose L, Nash M, Tan CEM. Engineered stochastic adhesion between microbes as a protection mechanism against environmental stress. Cell Mol Bioeng. 2018;11(5):367-82. |
| Li TT, Li CT, Butler K, Hays SG, Guarnieri MT, Oyler GA, et al. Mimicking lichens: incorporation of yeast strains together with sucrose-secreting cyanobacteria improves survival, growth, ROS removal, and lipid production in a stable mutualistic co-culture production platform. Biotechnol Biofuels. 2017;10:55. |
| Lindemann SR, Bernstein HC, Song HS, Fredrickson JK, Fields MW, Shou WY, et al. Engineering microbial consortia for controllable outputs. ISME J. 2016;10(9):2077-84. |
| Lockwood JL, Cassey P, Blackburn T. The role of propagule pressure in explaining species invasions. Trends Ecol Evol. 2005;20(5):223-8. |
| Loffler FE, Edwards EA. Harnessing microbial activities for environmental cleanup. Curr Opin Biotech. 2006;17(3):274-84. |
| Long RA, Rowley DC, Zamora E, Liu JY, Bartlett DH, Azam F. Antagonistic interactions among marine bacteria impede the proliferation of Vibrio cholerae. Appl Environ Microb. 2005;71(12):8531-6. |
| Lopez-Igual R, Bernal-Bayard J, Rodriguez-Paton A, Ghigo JM, Mazel D. Engineered toxin-intein antimicrobials can selectively target and kill antibiotic-resistant bacteria in mixed populations. Nat Biotechnol. 2019;37(7):755-60. |
| Maitner BS, Rudgers JA, Dunham AE, Whitney KD. Patterns of bird invasion are consistent with environmental filtering. Ecography. 2012;35(7):614-23. |
| Mallon CA, Poly F, Le Roux X, Marring I, van Elsas JD, Salles JF. Resource pulses can alleviate the biodiversity-invasion relationship in soil microbial communities. Ecology. 2015;96(4):915-26. |
| Maltz MR, Treseder KK. Sources of inocula influence mycorrhizal colonization of plants in restoration projects: a meta-analysis. Restor Ecol. 2015;23(5):625-34. |
| Manefield M, Whiteley A, Curtis T, Watanabe K. Influence of sustainability and immigration in assembling bacterial populations of known size and function. Microb Ecol. 2007;53(2):348-54. |
|  |
| Mansfeldt C, Achermann S, Men YJ, Walser JC, Villez K, Joss A, et al. Microbial residence time is a controlling parameter of the taxonomic composition and functional profile of microbial communities. ISME J. 2019;13(6):1589-601. |
| Markowiak P, Slizewska K. Effects of probiotics, prebiotics, and synbiotics on human health. Nutrients. 2017;9(9):1021. |
| Marsh P, Wellington EMH. Phage-host interactions in soil. FEMS Microbiol Ecol. 1994;15(1-2):99-107. |
| Matz C, Kjelleberg S. Off the hook - how bacteria survive protozoan grazing. Trends Microbiol. 2005;13(7):302-7. |
| Maynard DS, Crowther TW, Bradford MA. Competitive network determines the direction of the diversity-function relationship. Proc Natl Acad Sci USA. 2017;114(43):11464-9. |
| McNally L, Brown SP. Building the microbiome in health and disease: niche construction and social conflict in bacteria. Philos Trans R Soc B. 2015;370(1675):20140298. |
| Miller AD, Inamine H, Buckling A, Roxburgh SH, Shea K. How disturbance history alters invasion success: biotic legacies and regime change. Ecol Lett. 2021;24(4):687-97. |
| Mitchell CE, Agrawal AA, Bever JD, Gilbert GS, Hufbauer RA, Klironomos JN, et al. Biotic interactions and plant invasions. Ecol Lett. 2006;9(6):726-40. |
| Mitchell CE, Power AG. Release of invasive plants from fungal and viral pathogens. Nature. 2003;421(6923):625-7. |
| Muthukrishnan R, Hansel-Welch N, Larkin DJ. Environmental filtering and competitive exclusion drive biodiversity-invasibility relationships in shallow lake plant communities. J Ecol. 2018;106(5):2058-70. |
| Nakai T, Park SC. Bacteriophage therapy of infectious diseases in aquaculture. Res Microbiol. 2002;153(1):13-8. |
| Nale JY, Redgwell TA, Millard A, Clokie MRJ. Efficacy of an optimised bacteriophage cocktail to clear Clostridium difficile in a batch fermentation model. Antibiotics-Basel. 2018;7(1):13. |
|  |
| Nam IH, Hong HB, Kim YM, Kim BH, Murugesan K, Chang YS. Biological removal of polychlorinated dibenzo-p-dioxins from incinerator fly ash by Sphingomonas wittichii RW1. Water Res. 2005;39(19):4651-60. |
| Nguyen HT, Nguyen TT, Pham HTT, Nguyen QTN, Tran MT, Nguyen AH, et al. Fate of carotenoid-producing Bacillus aquimaris SH6 colour spores in shrimp gut and their dose-dependent probiotic activities. PloS One. 2018;13(12):e0209341. |
| Northfield TD, Laurance SGW, Mayfield MM, Paini DR, Snyder WE, Stouffer DB, et al. Native turncoats and indirect facilitation of species invasions. Proc Biol Sci. 2018;285(1871):20171936. |
| Nuttle T. Evaluation of restoration practice based on environmental filters. Restor Ecol. 2007;15(2):330-3. |
| O'Callaghan M. Microbial inoculation of seed for improved crop performance: issues and opportunities. Appl Microbiol Biotechnol. 2016;100(13):5729-46. |
| Otte MP, Gagnon J, Comeau Y, Matte N, Greer CW, Samson R. Activation of an indigenous microbial consortium for bioaugmentation of pentachlorophenol/creosote contaminated soils. Appl Microbiol Biotechnol. 1994;40(6):926-32. |
| Paine RT. Food web complexity and species diversity. Am Nat. 1966;100(910):65-75. |
| Palmer MA, Ambrose RF, Poff NL. Ecological theory and community restoration ecology. Restor Ecol. 1997;5(4):291-300. |
| Panigrahi P, Parida S, Nanda NC, Satpathy R, Pradhan L, Chandel DS, et al. A randomized synbiotic trial to prevent sepsis among infants in rural India (vol 548, pg 407, 2017). Nature. 2018;553(7687):238. |
| Pascual-Garcia A, Bonhoeffer S, Bell T. Metabolically cohesive microbial consortia and ecosystem functioning. Philos Trans R Soc B. 2020;375(1798):20190245. |
| Pereira FC, Berry D. Microbial nutrient niches in the gut. Environ Microbiol. 2017;19(4):1366-78. |
| Perez-Gutierrez RA, Lopez-Ramirez V, Islas A, Alcaraz LD, Hernandez-Gonzalez I, Olivera BCL, et al. Antagonism influences assembly of a Bacillus guild in a local community and is depicted as a food-chain network. ISME J. 2013;7(3):487-97. |
| Pernthaler J. Predation on prokaryotes in the water column and its ecological implications. Nat Rev Microbiol. 2005;3(7):537-46. |
| Perraudeau F, McMurdie P, Bullard J, Cheng AN, Cutcliffe C, Deo A, et al. Improvements to postprandial glucose control in subjects with type 2 diabetes: a multicenter, double blind, randomized placebo-controlled trial of a novel probiotic formulation. BMJ Open Diabetes Res Care. 2020;8(1). |
| Petschow BW, Figueroa R, Harris CL, Beck LB, Ziegler E, Goldin B. Effects of feeding an infant formula containing lactobacillus GG on the colonization of the intestine - A dose-response study in healthy infants. J Clin Gastroenterol. 2005;39(9):786-90. |
| Piccardi P, Vessman B, Mitri S. Toxicity drives facilitation between 4 bacterial species. Proc Natl Acad Sci USA. 2019;116(32):15979-84. |
| Pokusaeva K, Fitzgerald GF, van Sinderen D. Carbohydrate metabolism in Bifidobacteria. Genes Nutr. 2011;6(3):285-306. |
| Postma J, Hokahin CH, Vanveen JA. Role of microniches in protecting introduced Rhizobium-leguminosarum biovar Trifolii against competition and predation in soil. Appl Environ Microb. 1990;56(2):495-502. |
| Power B, Liu X, Germaine KJ, Ryan D, Brazil D, Dowling DN. Alginate beads as a storage, delivery and containment system for genetically modified PCB degrader and PCB biosensor derivatives of Pseudomonas fluorescens F113. J Appl Microbiol. 2011;110(5):1351-8. |
| Praveschotinunt P, Duraj-Thatte AM, Gelfat I, Bahl F, Chou DB, Joshi NS. Engineered E. coli Nissle 1917 for the delivery of matrix-tethered therapeutic domains to the gut. Nat Commun. 2019;10(1):5580. |
| Remans R, Croonenborghs A, Gutierrez RT, Michiels J, Vanderleyden J. Effects of plant growth-promoting rhizobacteria on nodulation of Phaseolus vulgaris L. are dependent on plant P nutrition. Eur J Plant Pathol. 2007;119(3):341-51. |
| Ripple WJ, Beschta RL. Trophic cascades in Yellowstone: The first 15 years after wolf reintroduction. Biol Conserv. 2012;145(1):205-13. |
| Ripple WJ, Estes JA, Beschta RL, Wilmers CC, Ritchie EG, Hebblewhite M, et al. Status and ecological effects of the world's largest carnivores. Science. 2014;343(6167):151. |
| Rivett DW, Jones ML, Ramoneda J, Mombrikotb SB, Ransome E, Bell T. Elevated success of multispecies bacterial invasions impacts community composition during ecological succession. Ecol Lett. 2018;21(4):516-24. |
| Russel J, Roder HL, Madsen JS, Burmolle M, Sorensen SJ. Antagonism correlates with metabolic similarity in diverse bacteria. Proc Natl Acad Sci USA. 2017;114(40):10684-8. |
| Safferman RS, Morris ME. Evaluation of natural products for algicidal properties. Appl Microbiol. 1962;10(4):289. |
| Sasse J, Martinoia E, Northen T. Feed your friends: do plant exudates shape the root microbiome? Trends in Plant Sci. 2018;23(1):25-41. |
| Schantz MC, Sheley RL, James JJ. Role of propagule pressure and priority effects on seedlings during invasion and restoration of shrub-steppe. Biol Invasions. 2015;17(1):73-85. |
| Seastedt TR, Pyšek P. Mechanisms of plant invasions of North American and European grasslands. Annu Rev Ecol Evol Syst. 2011;42(1):133-53. |
| Seekatz AM, Aas J, Gessert CE, Rubin TA, Saman DM, Bakken JS, et al. Recovery of the gut microbiome following fecal microbiota transplantation. Mbio. 2014;5(3). |
| Shade A, Peter H, Allison SD, Baho DL, Berga M, Burgmann H, et al. Fundamentals of microbial community resistance and resilience. Front Microbiol. 2012;3:417. |
| Shahab RL, Brethauer S, Luterbacher JS, Studer MH. Engineering of ecological niches to create stable artificial consortia for complex biotransformations. Curr Opin Biotech. 2020;62:129-36. |
| Shapiro OH, Kushmaro A, Brenner A. Bacteriophage predation regulates microbial abundance and diversity in a full-scale bioreactor treating industrial wastewater. ISME J. 2010;4(3):327-36. |
| Sharma S, Kanwar SS. Adherence potential of indigenous lactic acid bacterial isolates obtained from fermented foods of Western Himalayas to intestinal epithelial Caco-2 and HT-29 cell lines. J Food Sci Tech Mys. 2017;54(11):3504-11. |
| Shaw AJ, Lam FH, Hamilton M, Consiglio A, MacEwen K, Brevnova EE, et al. Metabolic engineering of microbial competitive advantage for industrial fermentation processes. Science. 2016;353(6299):583-6. |
| Shea K, Chesson P. Community ecology theory as a framework for biological invasions. Trends Ecol Evol. 2002;17(4):170-6. |
| Sheley RL, James JJ, Bard EC. Augmentative restoration: repairing damaged ecological processes during restoration of heterogeneous environments. Invas Plant Sci Mana. 2009;2(1):10-21. |
| Shepherd ES, DeLoache WC, Pruss KM, Whitaker WR, Sonnenburg JL. An exclusive metabolic niche enables strain engraftment in the gut microbiota. Nature. 2018;557(7705):434-38. |
| Simberloff D. The role of propagule pressure in biological invasions. Annu Rev Ecol Evol Syst. 2009;40:81-102. |
| Sinclair ARE, Mduma SAR, Hopcraft JGC, Fryxell JM, Hilborn R, Thirgood S. Long-term ecosystem dynamics in the Serengeti: Lessons for conservation. Conserv Biol. 2007;21(3):580-90. |
| Soundararajan M, von Bunau R, Oelschlaeger TA. K5 capsule and lipopolysaccharide are important in resistance to T4 phage attack in probiotic E. coli strain Nissle 1917. Front Microbiol. 2019;10:2783. |
| Sriswasdi S, Yang CC, Iwasaki W. Generalist species drive microbial dispersion and evolution. Nat Commun. 2017;8:1162. |
| Suez J, Zmora N, Zilberman-Schapira G, Mor U, Dori-Bachash M, Bashiardes S, et al. Post-antibiotic gut mucosal microbiome reconstitution Is Impaired by probiotics and improved by autologous FMT. Cell. 2018;174(6):1406-1423. |
| Thingstad TF. Elements of a theory for the mechanisms controlling abundance, diversity, and biogeochemical role of lytic bacterial viruses in aquatic systems. Limnol Oceanogr. 2000;45(6):1320-8. |
| Thompson IP, van der Gast CJ, Ciric L, Singer AC. Bioaugmentation for bioremediation: the challenge of strain selection. Environ Microbiol. 2005;7(7):909-15. |
| Tilman D. Niche tradeoffs, neutrality, and community structure: A stochastic theory of resource competition, invasion, and community assembly. Proc Natl Acad Sci USA. 2004;101(30):10854-61. |
| Tripathi S, Srivastava P, Devi R, Bhadouria R. Influence of synthetic fertilizers and pesticides on soil health and soil microbiology. In: Prasad MNV (ed). Agrochemicals detection, treatment and remediation. (Butterworth-Heinemann, 2020) pp 25-54. |
| Trosvik P, de Muinck EJ. Ecology of bacteria in the human gastrointestinal tract-identification of keystone and foundation taxa. Microbiome. 2015;3:44. |
| Umu OCO, Rudi K, Diep DB. Modulation of the gut microbiota by prebiotic fibres and bacteriocins. Microb Ecol Health Dis. 2017;28(1):1348886. |
| Upton RN, Bach EM, Hofmockel KS. Spatio-temporal microbial community dynamics within soil aggregates. Soil Biol Biochem. 2019;132:58-68. |
| Valdez SR, Zhang YS, van der Heide T, Vanderklift MA, Tarquinio F, Orth RJ, et al. Positive ecological interactions and the success of seagrass restoration. Front Mar Sci. 2020;7:91. |
| van Elsas JD, Chiurazzi M, Mallon CA, Elhottova D, Kristufek V, Salles JF. Microbial diversity determines the invasion of soil by a bacterial pathogen. Proc Natl Acad Sci U S A. 2012;109(4):1159-64. |
| Vasquez E, Sheley R, Svejcar T. Creating invasion resistant soils via nitrogen management. Invas Plant Sci Mana. 2008;1(3):304-14. |
| Vila JCC, Jones ML, Patel M, Bell T, Rosindell J. Uncovering the rules of microbial community invasions. Nat Ecol Evol. 2019;3(8):1162-1171. |
| Von Holle B, Simberloff D. Ecological resistance to biological invasion overwhelmed by propagule pressure. Ecology. 2005;86(12):3212-8. |
| Wainwright CE, Staples TL, Charles LS, Flanagan TC, Lai HR, Loy X, et al. Links between community ecology theory and ecological restoration are on the rise. J Appl Ecol. 2018;55(2):570-81. |
| Wang X, Wei Z, Yang K, Wang J, Jousset A, Xu Y, et al. Phage combination therapies for bacterial wilt disease in tomato. Nat Biotechnol. 2019;37(12):1513-20. |
| Wang XY, Cao ZP, Zhang MM, Meng L, Ming ZZ, Liu JY. Bioinspired oral delivery of gut microbiota by self-coating with biofilms. Sci Adv. 2020;6(26):eabb1952. |
| White EM, Wilson JC, Clarke AR. Biotic indirect effects: a neglected concept in invasion biology. Divers Distrib. 2006;12(4):443-55. |
| Wittmann MJ, Metzler D, Gabriel W, Jeschke JM. Decomposing propagule pressure: the effects of propagule size and propagule frequency on invasion success. Oikos. 2014;123(4):441-50. |
| Wright DA, Killham K, Glover LA, Prosser JI. Role of pore-size location in determining bacterial-activity during predation by protozoa in soil. Appl Environ Microb. 1995;61(10):3537-43. |
| Xing ZQ, Tang W, Geng WT, Zheng YN, Wang YP. In vitro and in vivo evaluation of the probiotic attributes of Lactobacillus kefiranofaciens XL10 isolated from Tibetan kefir grain. Appl Microbiol Biotechnol. 2017;101(6):2467-77. |
| Zampieri G, Vijayakumar S, Yaneske E, Angione C. Machine and deep learning meet genome-scale metabolic modeling. PLoS Comput Biol. 2019;15(7):e1007084. |
| Zapien-Campos R, Olmedo-Alvarez G, Santillan M. Antagonistic interactions are sufficient to explain self-assemblage of bacterial communities in a homogeneous environment: a computational modeling approach. Front Microbiol. 2015;6. |
| Zhang L, Chen L, Diao JJ, Song XY, Shi ML, Zhang WW. Construction and analysis of an artificial consortium based on the fast-growing cyanobacterium Synechococcus elongatus UTEX 2973 to produce the platform chemical 3-hydroxypropionic acid from CO2. Biotechnol Biofuels. 2020;13(1):82. |
| Zhang X, Ma J, Chen M, Wu Z, Wang Z. Microbial responses to transient shock loads of quaternary ammonium compounds with different length of alkyl chain in a membrane bioreactor. AMB Express. 2018;8(1):118. |
| Zhao D, Wu SG, Feng WW, Jakovlic I, Tran NT, Xiong F. Adhesion and colonization properties of potentially probiotic Bacillus paralicheniformis strain FA6 isolated from grass carp intestine. Fish Sci. 2020;86(1):153-61. |
| Zhao X, Wang W, Blaine A, Kane ST, Zijlstra RT, Ganzle MG. Impact of probiotic Lactobacillus sp. on autochthonous lactobacilli in weaned piglets. J Appl Microbiol. 2019;126(1):242-54. |
| Zhou JZ, Ning DL. Stochastic Community Assembly: Does It Matter in Microbial Ecology? Microbiol Mol Biol Rev. 2017;81(4). |
