# Supplementary material for: Solutions in microbiome engineering: prioritizing barriers to organism establishment
Source: ISME J. 2021 Aug 21;16(2):331–8. doi: 10.1038/s41396-021-01088-5 (PMC8776856; doi:10.1038/s41396-021-01088-5)
Supplement: Supplementary file 2 — Table S2 [file 41396_2021_1088_MOESM2_ESM.docx]

**Table S2.** Terminology across disciplines related to key concepts in microbiome engineering

| **Term** | **Field** | **Alternative Terms/Related Concepts** |
| --- | --- | --- |
| **Inoculant** | Human/animal microbiome | probiotics, microbiome transfers, live bio-therapeutic products, fecal transplants, colonizers, donor microbiome/strains |
|  | Agriculture/soil microbiome | inoculants, biocontrol agents, plant growth promoting microbes (PGPMs), probiotics, biofertilizer, biostimulant. bioprotectant, propagules, Immigrants |
|  | Wastewater / Drinking water treatment | inoculants, bioaugmentation, biostimulant |
|  | Bioremediation | bioaugmentation, biostimulant, propagule, target species |
|  | Invasion Biology | invaders, aliens, exotic species, non-native species, biocontrol agents |
|  | Restoration Ecology | colonists, focal species, recruits, priority species, desirable species, target species, seed mix |
| **Resident** | Human/animal microbiome | initial microbiome, recipient microbiome |
|  | Agriculture/soil microbiome | initial microbiome |
|  | Wastewater / Drinking water treatment | native community, strict colonizer, leaky colonizer, transcient colonizer, biofilm community |
|  | Bioremediation | native microbiome |
|  | Invasion Biology | native species |
|  | Restoration Ecology | endogenous species, seed community, native species, local species |
| **End Community** | Human/animal microbiome | healthy microbiome, reprogrammed community, final microbiome, altered microbiome |
|  | Agriculture/soil microbiome | final microbiome |
|  | Wastewater / Drinking water treatment | biofilm community, planktonic community |
|  | Bioremediation | bioaugmented community |
|  | Invasion Biology | invaded communiy, endpoint, altered community |
|  | Restoration Ecology | historical state, native state, endpoint, target community |
|  |  |  |
| **Propagule Pressure** | Human/animal microbiome | dose, frequency, delivery mode |
|  | Agriculture/soil microbiome | application amount, number of propagules, inoculant dose, frequency of application, delivery method |
|  | Wastewater / Drinking water treatment | concentration, relative abundance, symbiotic pressures, addition method, environmental factors, engineering parameters (e.g., flow rate, surface area to volume ratio etc) |
|  | Bioremediation | propagule density, enrichment methodology, |
|  | Invasion Biology | dispersal rate, regional factors, species transport |
|  | Restoration Ecology | dispersal rate, regional factors, species transport, propagule delivery |
